# Frequency of drug-resistant bacterial isolates among pregnant women with UTI in maternity and children’s hospital, Bisha, Saudi Arabia

**DOI:** 10.1038/s41598-024-58275-5

**Published:** 2024-03-28

**Authors:** Ghady S. Al-Shahrani, Tareg M. Belali

**Affiliations:** Faculty of Applied Medical Sciences, University of Bisha, 255, Al Nakhil, 67714 Bisha, Saudi Arabia

**Keywords:** Urinary tract infections, Complications, Prevalence, Antimicrobial susceptibility, Pyelonephritis, Microbiology, Health care

## Abstract

Urinary tract infections (UTIs) are one of the most prevalent bacterial infections affecting humans, with a higher incidence among women. Pregnant women are at an increased risk of developing UTIs, which can have detrimental consequences for both the mother and fetus. UTIs can be caused by various bacteria, and the prevalence of drug-resistant UTIs in maternity and children’s hospitals is a cause for concern due to the potential for severe complications if left untreated. The primary objective of the current study was to determine the distribution of UTI-causing bacteria and investigate the antibiotic sensitivity patterns of isolated cultures obtained from pregnant women with UTIs at the Maternity and Children’s Hospital, Bisha, Saudi Arabia. This cross-sectional study was conducted from October 2021 to October 2023, involving the analysis of urine samples collected from 321 participants who acquired UTIs during pregnancy. Using biochemical tests and standard cultures, the urine samples were examined for pathogenic bacteria and their anti-microbial sensitivity patterns. The study analyzed susceptibility results according to the Clinical Laboratory Standards Institute guidelines (M100, 28th Edition, 2018). Bacterial strains demonstrating resistance to three or more antibiotics were classified as multidrug-resistant (MDR). This study revealed the distribution of UTI-causing bacteria to be as follows: *Escherichia coli*, 57.01%; *Klebsiella pneumoniae,* 24.61%; *Pseudomonas aeruginosa*, 4.36%; *Proteus mirabilis* and *Enterobacter cloacae,* 3.74%; *Streptococcus agalactiae,* 3.11%; *Enterococcus faecalis,* 2.18%; and *Staphylococcus aureus,* 1.24%. Antimicrobial susceptibility testing varied among gram-positive and gram-negative bacteria. Gentamicin demonstrated the highest sensitivity among both gram-positive and gram-negative bacteria; piperacillin-tazobactam was the second most effective drug against gram-negative bacteria. The bacterial isolates showed varying susceptibility to different antibiotics, with *Escherichia coli*, *Klebsiella pneumoniae,* and *Pseudomonas aeruginosa* being mainly sensitive to gentamicin, piperacillin-tazobactam, and ciprofloxacin, respectively. The strategies for reducing the risk of UTIs need to be improved to limit the spread of MDR bacteria. These strategies may include promoting hygienic practices and administering appropriate antibiotics to prevent the emergence and spread of drug-resistant bacteria. Further research is required to monitor the trends in antibiotic resistance among UTI-causing bacteria and develop effective strategies for managing this public health menace.

## Introduction

Urinary tract infections (UTIs) are a common reason for individuals seeking medical attention at doctors’ offices and emergency rooms worldwide. In the United States alone, UTI-related symptoms account for about 10 million doctor visits annually^1^. UTIs primarily affect the bladder and urethra, causing discomfort if left untreated and potentially leading to severe health issues such as pyelonephritis, sepsis, and renal abscesses if the infections spreads to the kidneys^2–10^. Moreover, UTIs are particularly prevalent in pregnant women. Females exhibit higher susceptibility to UTIs compared to males due to anatomical factors such as a shorter urethra, lack of prostatic secretion, pregnancy, and a heightened risk of urinary tract contamination by fecal flora^11^. It is estimated that approximately 50% of women will experience a UTI at least once during adulthood, including during pregnancy^12^. In pregnant women, the incidence of UTIs is notably higher, affecting about 20% of individuals, constituting a significant health concern and often resulting in hospital admissions to obstetrical wards^13^. If left untreated, UTIs during pregnancy can lead to severe complications for both the mother and fetus, including low birth weight, preterm labor, fetal demise, and maternal complications such as anemia, preeclampsia, and septicemia^14^. Various pathogenic microorganisms, including bacteria, fungi, protozoa, and viruses, contribute to the occurrence of UTIs in pregnant women. Among these pathogens are *Escherichia coli*, *Staphylococcus aureus,* and *Klebsiella pneumoniae*^12^.

The prevalence of microbial illnesses has increased significantly in recent decades due to the overuse and inappropriate use of antimicrobial medications, leading to the emergence of resistance among different strains of microbes^15^. Multi-drug resistance is defined as a microorganism’s insensitivity or resistance to antimicrobial drugs, notwithstanding its previous sensitivity to them^16^. Multidrug resistant (MDR), extensively drug-resistant (XDR), and pan drug-resistant (PDR) bacterial strains, both pathogenic and opportunistic, pose considerable challenges in healthcare settings due to the limited availability of effective treatments^17^. The global prevalence of MDR bacteria, including highly resistant gram-negative strains like MDR carbapenemase-producing *Klebsiella pneumoniae* and *Acinetobacter* spp. varies significantly across regions. Similarly, the incidence of XDR bacteria, such as *Acinetobacter baumannii*, is increasing, especially in intensive care units. Although occurrences of PDR isolates like carbapenem-resistant *Enterobacteriaceae* have been documented in recent years, precise data remain limited^17^. The World Health Organization (WHO) warns that resilient bacteria can withstand antimicrobial drugs, leading to ineffective treatment, persistent infections, and further spread. Antimicrobial resistance is a global health concern, causing increased healthcare costs, prolonged hospital stays, and higher mortality rates^18^. Almost all major infectious agents encountered in clinical practice show substantial levels of resistance to common antibiotics, with many organisms classified as MDR^19^. The resistance of MDR, XDR, and PDR bacteria to antimicrobial medications has become a significant global public health threat. This rapid emergence of resistance mechanisms reduces the effectiveness of traditional treatments for common infectious diseases, resulting in prolonged illness durations, increased healthcare expenses, and higher mortality rates. According to WHO, bacteria such as *Escherichia coli, Klebsiella pneumoniae, Staphylococcus aureus, Streptococcus pneumoniae, Nontyphoidal Salmonella, and Shigella* species display high rates of resistance to various antibiotics, exacerbating the challenge of combating infectious diseases^20^. Understanding the pathogenic bacteria associated with UTIs in pregnant women and their antibiotic sensitivity is vital for implementing preventive measures in healthcare facilities. While numerous studies have investigated UTIs in different regions of Saudi Arabia, few have specifically focused on pregnant women in the Bisha area. Additionally, UTIs are influenced by different variables. Therefore, this study was conducted to evaluate the prevalence of pathogenic bacteria causing UTIs and their antibiotic susceptibility among pregnant women admitted to the Maternity and Children’s Hospital in Bisha, located in the Northern Asir region of Saudi Arabia.

## Methodology

### Sample size, inclusion criteria, exclusion criteria, and urine sample collection

This cross-sectional study was conducted at the Maternity and Children’s Hospital in Bisha, Saudi Arabia, from October 2021 to October 2023. The sample size of pregnant women was determined through a sample size calculation for proportions formula. This involved considering parameters such as a 50% prevalence rate of UTI, a margin of error (*d*) of 0.05, and (Zα/2) of 95% confidence interval (CI) level. The final sample size was 321.The study included pregnant women with confirmed UTIs who were on follow-up visits at the hospital’s antenatal care unit. Pregnant women who had taken antibiotics within 2 weeks prior to their hospital visit were excluded from the study. The study was conducted in accordance with the Declaration of Helsinki and after meeting the ethical standards set by Bisha University. A total of 321 clean-catch midstream urine samples were collected in wide-mouth sterile containers, appropriately labeled, and promptly transported to the microbiology laboratory at the Department of Medical Laboratory Sciences, University of Bisha, within 1 h. Bacterial uropathogens were isolated, and their patterns of antimicrobial drug resistance were assessed. All participants voluntarily took part in this study and provided written informed consent after meeting the ethical standards for participation set forth by the research protocol.

### Isolation and identification of bacterial colonies

Using a calibrated inculcation loop (0.001 ml), the urine samples were cultivated in standard culture media, including 5% sheep blood agar, MacConkey agar, LB Agar, and Pseudosel agar (Fisher Scientific, US). A urine specimen was deemed positive for a UTI if, following a two-day incubation period at 37 °C, a single organism was cultured at a concentration of ≥ 10^5^ colony-forming units per milliliter (cfu/ml).

Positive cultures exhibiting significant bacteriuria were subsequently identified at the species level based on colony characteristics, gram-staining reaction, and biochemical profiles assessed using standard biochemical tests. These tests included indole, citrate, oxidase, hydrogen sulfide (H2S) production, lysine decarboxylase, lactose fermentation, urea hydrolysis, gas production, catalase, coagulase, mannitol fermentation, and novobiocin susceptibility tests.

Quality control parameters for laboratory tests included reference strains from the American Type Culture Collection (ATCC) such as *Escherichia coli* (ATCC-25922), *Proteus mirabilis* (ATCC-29906), *Pseudomonas aeruginosa* (ATCC-27853), and *Enterococcus faecalis* (ATCC-29212).

### Antibiotic sensitivity testing

The isolates’ antimicrobial susceptibility was evaluated using the disk diffusion technique. The surface of the Mueller–Hinton agar was uniformly covered with standard inoculum adjusted to 0.5 McFarland standard turbidity (Fisher Scientific, US). Antimicrobial disks containing (Fisher Scientific, US) 35 µg each of ampicillin, amoxicillin, clavulanate, cefazolin, ceftriaxone, and ciprofloxacin, 20 µg each of vancomycin, piperacillin-tazobactam, nitrofurantoin, meropenem, and cefuroxime, and 15 µg of gentamicin were placed on the Mueller–Hinton agar plates. The zone of inhibition was determined after incubating overnight at 37 °C, and according to the established criteria, bacterial antibiotic sensitivity was classified as sensitive, intermediately sensitive, or resistant. The antimicrobials were procured from Oxoid Ltd. in Basingstoke Hampshire, UK. A standard inoculum adjusted to 0.5 McFarland was applied to Mueller–Hinton agar from the same supplier. Antibiotic discs were then dispensed onto the dried plates and incubated at 37 °C for 24 h. The susceptibility findings were analyzed in line with the Clinical Laboratory Standards Institute guidelines (M100, 28th Edition, 2018). Bacterial strains demonstrating resistance to three or more antibiotics were categorized as MDR.

MDR organisms are labeled as such due to their resistance to one or more antimicrobial agents in laboratory tests. Conversely, XDR bacteria are of particular concern because of their resistance to multiple antimicrobial agents. The term “XDR” was initially coined to describe *Mycobacterium tuberculosis* strains exhibiting extensive drug resistance. Additionally, pan drug-resistance refers to bacteria that are resistant to every available antimicrobial agent^17,21^.

### Statistics and data analyses

Categorical variables were summarized, with numerical and percentage data presented in tables created using Microsoft Excel. Prism GraphPad was used to create graphical representations of bacterial prevalence, and statistical differences between the antibiotic susceptibility results were assessed using the chi-square test, with a significance level of p < 0.05 and a 95% confidence interval.

### Ethics approval

The university of Bisha ethics committee reviewed and approved the ethical criteria of this study.

## Results

### Distribution of UTI bacteria among pregnant women

**Figure 1:**
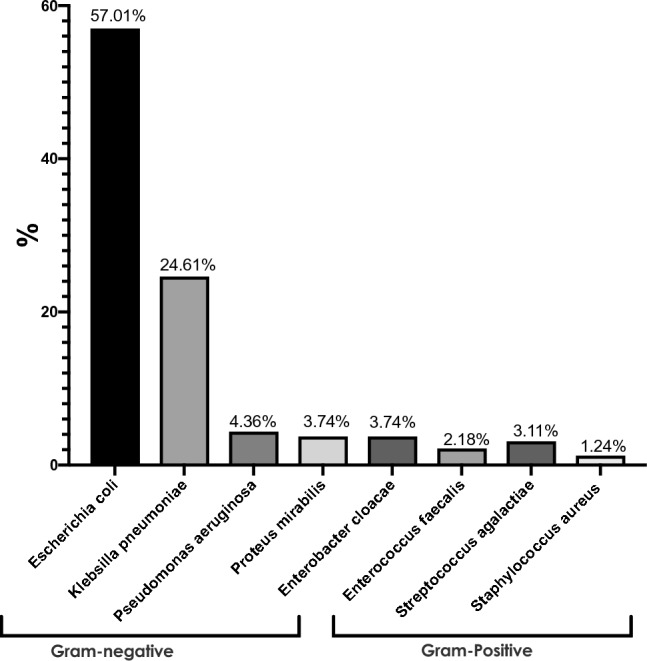
Prevalence of Bacteria Isolated from cultures of urine samples of pregnant women with UTIs at the maternity and children’s hospital, Bisha, Saudi Arabia.

A total of 321 pregnant women aged 18 to 48 years were recruited for this study (Table 1). The most prevalent pathogen isolated from pregnant women with UTIs admitted to the Maternity and Children’s Hospital was *Escherichia coli*—183 (57.01%), followed by *Klebsiella pneumoniae*—79 (24.61%), and then *Pseudomonas aeruginosa*—14 (4.36%), *Proteus mirabilis* and *Enterobacter cloacae*—12 (3.74%), *Streptococcus agalactiae*—10 (3.11%), and *Enterococcus faecalis*—7 (2.18%). *Staphylococcus aureus* was the least prevalent pathogen among the participants—4 (1.24%) (Fig. 1).

**Table 1:** Age distribution of pregnant women with UTIs at the maternity and children's hospital, Bisha, Saudi Arabia.

Age group	Number of participants
18–25	61
26–40	214
41–48	46

### Antibiotic susceptibility pattern among pregnant women with UTIs

Gentamicin demonstrated the highest percentage of antibiotic sensitivity among gram-positive bacteria (76.1%), followed by ceftriaxone (38.09%), meropenem, vancomycin, and cefuroxime (33.3%), nitrofurantoin (23.8%), ampicillin and ciprofloxacin (19.04%), and cefazolin (4.7%). In terms of specific bacterial isolates, *Streptococcus agalactiae* exhibited the highest antimicrobial susceptibility to gentamicin (100%), followed by ceftriaxone (80%), meropenem and cefuroxime (70%), and ampicillin (20%). *Enterococcus faecalis* was most sensitive to vancomycin (71.4%), followed by gentamicin and ciprofloxacin (42.8%), and then ampicillin and nitrofurantoin (28.5%). *Staphylococcus aureus* revealed the highest sensitivity to gentamicin and nitrofurantoin (75%), followed by vancomycin (50%) and cefazolin and ciprofloxacin (25%). Although some gram-positive uropathogens displayed varying sensitivity to certain antibiotics, the chi-square test indicated statistically significant variation only for gentamicin and ceftriaxone (****P < 0.0001) (Table 2).

**Table 2:** Antibiotic susceptibility patterns of gram-positive uti-causing bacteria in pregnant women at the maternity and children’s hospital, Bisha, Saudi Arabia.

Isolates	Pattern	Antibiotics (%)								
		GEN	CEF	MEM	VAN	CXM	NFT	AMP	CIP	CZO
*E. faecalis* (N = 7)	S	3 (42.8%)			5 (71.4%)		2 (28.5%)	2 (28.5%)	3 (42.8%)	
	R	4 (57.1%)			2 (28.5%)		5 (71.4%)	5 (71.4%)	4 (57.1%)	
*S. aureus* (N = 4)	S	3 (75%)			2 (50%)		3 (75%)		1 (25%)	1 (25%)
	R	1 (25%)			2 (50%)		1 (25%)		3 (75%)	3 (75%)
*S. agalactiae* (N = 10)	S	10 (100%)	8 (80%)	7 (70%)		7 (70%)		2 (20%)		
	R		2 (20%)	3 (30%)		3 (30%)		8 (70%)		
Total (N = 21)	S	16 (76.1%)	8 (38.09%)	7 (33.3%)	7 (33.3%)	7 (33.3%)	5 (23.8%)	4 (19.04%)	4 (19.04%)	1 (4.7%)
	R	5 (23.8%)	2 (9.5%)	3 (14.2%)	4 (19.04%)	3 (14.2%)	6 (28.5%)	13 (61.9%)	7 (33.33%)	3 (14.2%)
*p* value	< 0.0001		< 0.0001	0.112	0.355	0.112	0.5166	0.533	0.355	0.238

*GEN* gentamicin, *CEF* ceftriaxone, *MER* meropenem, *VAN* vancomycin, *CXM* cefuroxime, *NFT* nitrofurantoin, *AMP* ampicillin, *CIP* ciprofloxacin, *CZO* cefazolin, *S, R* sensitive, resistance.

Among gram-negative bacteria, gentamicin had the highest antibiotic sensitivity at 46%, followed by piperacillin-tazobactam (45.6%), ciprofloxacin (35.3%), nitrofurantoin (27.3%), ampicillin (25%), ceftriaxone (19.3%), and cefazolin (18.3%). The lowest antibiotic sensitivity among gram-negative bacteria was observed for amoxicillin-clavulanate (14.3%). Regarding specific bacterial isolates, the antimicrobial susceptibility for *Escherichia coli* was highest for piperacillin-tazobactam (52.4%), followed by gentamicin (50.8%), ciprofloxacin (36.6%), ampicillin (32.2%), ceftriaxone (27.8%), nitrofurantoin (26.7%), cefazolin (25.1%), and amoxicillin-clavulanate (15.3%).

*Klebsiella pneumoniae* displayed the highest antimicrobial susceptibility to gentamicin (34.1%) and piperacillin-tazobactam (32.9%), followed by ceftolozane-tazobactam and nitrofurantoin (26.5%), and ampicillin (17.7%). *Pseudomonas aeruginosa* exhibited the highest susceptibility to ciprofloxacin (71.4%), followed by piperacillin-tazobactam (57.1%) and gentamicin (42.8%). For *Proteus mirabilis*, the antimicrobial susceptibility percentages were highest for piperacillin-tazobactam (58.3%), followed by gentamicin (50%), ampicillin, ceftriaxone, and cefazolin (33.3%), and amoxicillin-clavulanate (25%). Finally, *Enterobacter cloacae* showed the highest antimicrobial susceptibility to amoxicillin-clavulanate and nitrofurantoin (100%), followed by ciprofloxacin (58.3%), gentamicin (50%), cefazolin (41.6%), and ceftriaxone (25%). While some gram-negative uropathogens displayed varying sensitivity to certain antibiotics, the chi-square test revealed statistically significant variation only for gentamicin and piperacillin-tazobactam (****P < 0.0001) (Table 3).

**Table 3:** Antibiotic susceptibility patterns of gram-negative bacteria causing UTIs in pregnant women at the maternity and children’s hospital, Bisha, Saudi Arabia.

Isolates	Pattern	Antibiotics (%)							
		GEN	TZP	CIP	NFT	AMP	CEF	CZO	AMK
*E. coli* (N = 183)	S	93 (50.8%)	96 (52.4%)	67 (36.6%)	49 (26.7%)	59 (32.2%)	51 (27.8%)	46 (25.1%)	28 (15.3)
	R	144 (78.6%)	87 (47.5%)	116 (63.3%)	134 (73.2%)	124 (67.7%)	132 (72.1%)	137 (74.8%)	155 (84.6%)
*K. pneumoniae* (N = 79)	S	27 (34.1%)	26 (32.9%)	21 (26.5%)	21 (26.5%)	14 (17.7%)			
	R	52 (65.8%)	53 (67.0%)	58 (73.4%)	58 (73.4%)	65 (82.2%)			
*P. aeruginosa* (N = 14)	S	6 (42.8%)	8 (57.1%)	10 (71.4%)					
	R	8 (57.1%)	6 (42.8%)	4 (28.5%)					
*P. mirabilis* (N = 12)	S	6 (50%)	7 (58.3%)	1 (8.3%)		4 (33.3%)	4 (33.3%)	4 (33.3%)	3 (25%)
	R	6 (50%)	5 (41.6%)	11 (91.6%)		8 (66.6%)	8 (66.6%)	8 (66.6%)	9 (75%)
*E. cloacae* (N = 12)	S	6 (50%)		7 (58.3%)	12 (100%)		3 (25%)	5 (41.6%)	12 (100%)
	R	6 (50%)		5 (41.6%)			9 (75%)	7 (58.3%)	
Total (N = 300)	S	138 (46%)	137 (45.6%)	106 (35.3%)	82 (27.3%)	77 (25%)	58 (19.3%)	55 (18.3%)	43 (14.3%)
	R	216 (72%)	151 (50.3%)	194 (64.6)	192 (64%)	197 (65.6%)	194 (64.6%)	152 (50.6%)	164 (54.6%)
*p* value		< 0.0001	< 0.0001	0.143	0.211	0.513	0.176	0.156	0.896

*GEN* gentamicin, *TZP* piperacillin-tazobactam, *CIP* ciprofloxacin, *NFT* nitrofurantoin, *AMP* ampicillin, *CEF* ceftriaxone, *CZO* cefazolin, *AMK* amoxicillin-clavulanate, *S, R* sensitive, resistance.

## Discussion

UTIs are among the most common types of infections, particularly prevalent among pregnant women compared to non-pregnant women. This poses a significant health risk, often requiring hospitalization in obstetrical units. Inefficient treatment of UTIs can lead to severe complications for both the mother and fetus, including low birth weight, intrauterine growth restriction, premature labor, early delivery, fetal demise, and higher rates of prenatal mortality and morbidity.

typical for UTIs, with the choice of antimicrobial drug based on the most likely infection and its anticipated antimicrobial resistance pattern in a specific area^10^. The present study revealed the incidence of antimicrobial resistance among bacteria isolated from pregnant women with UTIs in the Maternity and Children’s Hospital in Bisha, Kingdom of Saudi Arabia.

The unique structures of gram-negative bacteria help them adhere to the surface of uroepithelial cells, preventing their removal through urinary lavage. This attachment allows for bacterial multiplication and tissue invasion, ultimately leading to invasive infections like pyelonephritis during pregnancy. Moreover, the incidence of UTIs due to gram-negative uropathogens is higher than that of gram-positive bacteria. Our results indicate that gram-negative bacteria isolates were more prevalent at 93.4%, compared to gram-positive bacteria isolates at 6.5%. A similar trend was observed in Addis Ababa, the capital of Ethiopia, where 68.4% of isolates were gram-negative and 31.6% were gram-positive bacteria^22^. Similarly, in Riyadh, Saudi Arabia, gram-negative bacteria accounted for 91.98% of isolates, while gram-positive bacteria constituted 6.79%^23^. In the distribution of infectious organisms among patients with UTIs, the current study has identified *Escherichia coli* (57.01%) as the most common bacterium isolated in pregnant women, followed by *Klebsiella pneumoniae* (24.61%). These findings align with previous research studies conducted in various countries^23,24^, including Saudi Arabia^22,25^, where *Escherichia coli* and *Klebsiella pneumoniae* were identified as the most common causative uropathogens in UTI cases. The presence of isolate *Proteus mirabilis* was also detected in the current study’s participants, ranking third at 3.74%. This discovery aligns with previous research conducted by Kazmi et al. and Ahmad in Saudi Arabia^26,27^. However, our findings contrast with a separate study where *Proteus mirabilis* ranked fifth^28^. There are several potential reasons for these variations. First, our study specifically targeted hospitalized patients, who are more susceptible to *Proteus mirabilis* infections, particularly following urethral catheterization. Second, there were variations in the demographics of our participants, as our research focused on pregnant women, who face an increased risk of recurrent UTIs and subsequent *Proteus* infections.

The results of this study revealed that gram-negative bacteria exhibited varying levels of antimicrobial susceptibility, with rates ranging from 14.3% (for amoxicillin-clavulanate) to 46% (for gentamicin) and 45.6% (for piperacillin-tazobactam). In contrast, gram-negative bacteria displayed a low sensitivity to ampicillin (25%), ceftriaxone (19.3%), and nitrofurantoin (27.3%). Unsurprisingly, a low sensitivity pattern was also observed across most tested drugs, including amoxicillin-clavulanate and, to a lesser extent, cefazolin and others. Although gram-negative and gram-positive uropathogens demonstrated diverse sensitivities to specific antibiotics, the chi-square test only indicated statistically significant differences for gentamicin and ceftriaxone (****P < 0.0001) As seen in Table 3, gram-negative bacteria (*Escherichia coli* and *Klebsiella pneumoniae*) have low sensitivity to ciprofloxacin and ampicillin. These findings align with those of other studies conducted in Saudi Arabia^29,30^, Europe, Canada, and Africa^31–33^ that underscored the high resistance of urinary pathogens to commonly used antibiotics such as piperacillin-tazobactam and ampicillin. Despite the widespread empirical use of ciprofloxacin for treating UTIs, the current study revealed that only 35.3% and 19.04% of gram-negative and gram-positive bacteria, respectively, were sensitive to this medication (Tables 2, 3). These numbers are among the highest to be documented for single uropathogens. Conversely, *Proteus mirabilis*, *Klebsiella pneumoniae*, and *Pseudomonas aeruginosa* exhibited the lowest rates of drug sensitivity at 8.3%, 17.7%, and 42.8%, respectively. These findings challenge previous clinical guidelines and recommendations suggesting ciprofloxacin as the first-line treatment for UTIs^34,35^. Fluoroquinolones are another class of antibiotics that are frequently used to treat UTIs. In our study, the isolates’ rates of sensitivity to ciprofloxacin were 36.6% for *Escherichia coli*, 26.5% for *Klebsiella pneumoniae*, and 8.3% for *Proteus mirabilis*. These rates are comparable to those reported by Choe et al.^36^, who likewise noted a very high resistance rate to fluoroquinolones among uropathogens isolated from several Asian nations, with a sensitivity rate of 46.1% for ciprofloxacin. However, our results show significantly higher sensitivity rates than those seen in many recent studies conducted in Saudi Arabia as well as other European and North American nations^37^. Renal transplant, which was recently recognized as a separate risk factor for ciprofloxacin-resistant *Escherichia coli*, and prior exposure to fluoroquinolones are likely to be responsible for these low sensitivity levels^38^. Interestingly, the current study discovered that *Escherichia coli*, *Klebsiella pneumoniae*, and *Pseudomonas aeruginosa* isolates had the highest sensitivity to aminoglycosides (such as gentamicin), with rates of 50.8%, 34.1%, and 42.8%, respectively (Table 3). These findings align with previous research by Kalal and Nagaraj^39^ and Choe et al.^36^. *Escherichia coli* demonstrated the highest rates of susceptibility to gentamicin. Sensitivity to β-lactam/β-lactamase inhibitor combinations (piperacillin-tazobactam) was also observed in our investigation, with *Klebsiella pneumoniae* and *Escherichia coli* exhibiting sensitivity rates of 32.9% and 52.4%, respectively. *Pseudomonas isolates* also exhibited sensitivity to piperacillin-tazobactam at a rate of 57.1%. However, *Proteus* isolates had the highest sensitive to piperacillin-tazobactam (58.3%). These findings support earlier studies indicating that *Escherichia coli* exhibited the lowest level of resistance among piperacillin-tazobactam-resistant uropathogens^40^. Accordingly, it can be hypothesized that aminoglycosides (like gentamicin) are the first-line empirical treatment for UTIs in Bisha, followed by β-lactam/β-lactamase inhibitor combinations as an alternative second-line treatment. *Streptococcus agalactiae*, *Enterococcus faecalis*, and *Staphylococcus aureus* were the gram-positive uropathogens isolated in this study. These results are consistent with studies conducted in different regions of Saudi Arabia, including Al-Baha (the southern region of KSA) and Ha’il (the north-western region of KSA)^41^, where strains of these species were found to be typical UTI-causing bacteria. Gram-positive bacteria indicated low sensitivity to many of the tested antibiotics, including nitrofurantoin and ampicillin, likely due to the excessive use of antibiotics in this area. Another possible factor contributing to antibiotic resistance is drug abuse among the general public, with patients with various infections regularly self-medicating with antibiotics. Majority of the tested gram-positive uropathogens in this study showed the highest level of sensitivity to gentamicin, followed by ceftriaxone, meropenem, vancomycin, cefuroxime, and ciprofloxacin. The findings of the current study are consistent with those of Bitew et al.^22^, who revealed that gram-positive bacteria are sensitive to vancomycin, ciprofloxacin, and gentamicin. It is thus suggested that vancomycin and gentamicin be the first-line drugs for treating patients with UTIs caused by gram-positive bacteria in Bisha.

## Conclusion

*Escherichia coli* and *Klebsiella pneumoniae* were the most common uropathogens in UTI patients, with many gram-negative bacteria showing low sensitivity to amoxicillin-clavulanate. Gentamicin, ceftriaxone, and piperacillin-tazobactam were identified as highly effective drugs for the treatment of these pathogens. Gram-positive bacteria were susceptible to vancomycin, gentamicin, and ceftriaxone. These results highlight the necessity of switching out the first-line antibiotics currently in use with more potent substitutes. Antimicrobial sensitivity testing is highly recommended before prescribing antibiotics to prevent and slow the spread of antimicrobial resistance.

## Data Availability

The authors confirm that the data that support the findings of this study is available within the article and/or its Supplementary Materials.
